# Characterization of the complete chloroplast genome of the perennial plant *Tradescantia ohiensis* Raf. (Commelinales: Commelinaceae)

**DOI:** 10.1080/23802359.2021.2005477

**Published:** 2021-11-29

**Authors:** Yang Liu, Guang-Chen Liu, Ye Xu

**Affiliations:** School of Basic Medical Sciences, Jilin Medical University, Jilin, People’s Republic of China

**Keywords:** Bayesian inference, iterative mapping, phylogeny, plastid genome, spiderwort

## Abstract

*Tradescantia ohiensis* Raf. (Ohio spiderwort/blue-jacket) is a perennial herb native to North America that has become widely established in China. The chloroplast (cp) genome of *T. ohiensis* was assembled using Illumina sequencing reads. It is 164,140 bp in length with an A + T-biased nucleotide composition, and comprises a large single-copy (LSC) region (91,248 bp), a small single-copy (SSC) region (18,426 bp), and a pair of inverted repeat (IR) regions (27,233 bp). The cp genome harbors a total of 112 gene species with 19 of them being completely or partially duplicated and 18 of them possessing one or two introns. Phylogenetic analysis suggests that *T. ohiensis* is most closely related to the congeneric *T. virginiana*.

*Tradescantia ohiensis* Raf., commonly known as Ohio spiderwort or blue-jacket, is a perennial herb within the family Commelinaceae (order Commelinales) and is native to Eastern and Central North America (Hance et al. 2000). It is first introduced into China as an ornamental plant and later has become established naturally in many provinces of China (Z.-Q. Qian, pers. comm.). In the present study, we characterized the complete chloroplast (cp) genome for this exotic plant, and further investigated its phylogenetic placement.

Fresh leaves of *T. ohiensis* were collected from a single individual in Ningshan County, Shaanxi Province, China (108.55°E, 33.55°N), and were stored in alcohol for the subsequent DNA isolation. A specimen was deposited at the Herbarium of the School of Basic Medical Sciences, Jilin Medical University (http://www.jlmu.cn/; Ye Xu, Email: yl92@mail.ustc.edu.cn) under the voucher number TOHIE-2020-12-08. Total genomic DNA was extracted using the DNeasy Plant Mini Kit (Qiagen, CA, USA). High-throughput DNA sequencing was conducted by Novogene Bioinformatics Technology Company (Beijing, China) on the Illumina HiSeq X Ten Sequencing System (Illumina, CA, USA). Totally, 21.51 M of 150-bp paired-end reads were generated. The cp genome was assembled using the program MITObim v1.9 (Hahn et al. 2013) with that of *Hanguana malayana* (Jack) Merr. (GenBank accession no.: KT312930) (Barrett et al. 2016) as the initial reference. Annotation of the cp genome was conducted by comparing with those of phylogenetically related taxa.

The chloroplast genome of *T. ohiensis* is 164,140 bp in size, comprising a large single-copy (LSC) region (91,248 bp), a small single-copy (SSC) region (18,426 bp), and a pair of inverted repeat (IR) regions (27,233 bp). The nucleotide composition is asymmetric (31.7%A, 18.2%C, 17.6%G, and 32.5%) with an overall A + T content of 64.2% ('light strand'). The SSC, LSC, and IR regions differ obviously in nucleotide composition, with their A + T contents being 67.0%, 62.8%, and 57.7%, respectively. The cp genome harbors a total of 112 gene species, including 78 protein-coding (PCG), 30 tRNA, and four rRNA gene species. In all, 19 gene species are completely or partially duplicated, including eight PCGs (*ndh*B, *rpl*2, *rpl*23, *rps*7, *rps*12, *rps*19, *ycf*1, and *ycf*2), seven tRNAs (*trn*A-UGC, *trn*H-GUG, *trn*I-CAU, *trn*I-GAU, *trn*N-GUU, *trn*R-ACG, and *trn*V-GAC) and all four rRNAs (4.5S, 5S, 16S, and 23S rRNA). Ten PCGs (*atp*F, *ndh*A, *ndh*B, *pet*B, *pet*D, *rpl*2, *rpl*16, *rpo*C1, *rps*12, and *rps*16) and six tRNA gene species (*trn*A-UGC, *trn*G-UCC, *trn*I-GAU, *trn*K-UUU, *trn*L-UAA, and *trn*V-UAC) harbor a single intron, and two introns are detected in two protein-coding genes (*clp*P and *ycf*3).

A phylogenetic tree was reconstructed based on the Bayesian analysis of chloroplast PCGs for a panel of 18 taxa within Commelinaceae using the software MrBayes v3.1.1 (Huelsenbeck and Ronquist 2001; Ronquist and Huelsenbeck 2003) (Figure 1). As suggested by the ‘Model Selection’ function of the software TOPALi v2.5 (Milne et al. 2009), <GTR + G+I > was employed as the best-fit nucleotide substitution. *Hanguana malayana* (Jack) Merr. (KT312930; Commelinales: Hanguanaceae) (Barrett et al. 2016) was used as the outgroup taxon. As anticipated, all three species within the genus *Tradescantia* (i.e. *T. ohiensis*, *T. pallida,* and *T. virginiana*) were clustered together. Furthermore, *T. ohiensis* was closely related to *T. virginiana* than to *T. pallida*. This is not strange, since *T. ohiensis* and *T. virginiana* are taxonomically placed within the section Tradescantia while *T. pallida* belongs to the section Setcreasea (Burns et al. 2011).

**Figure 1.**
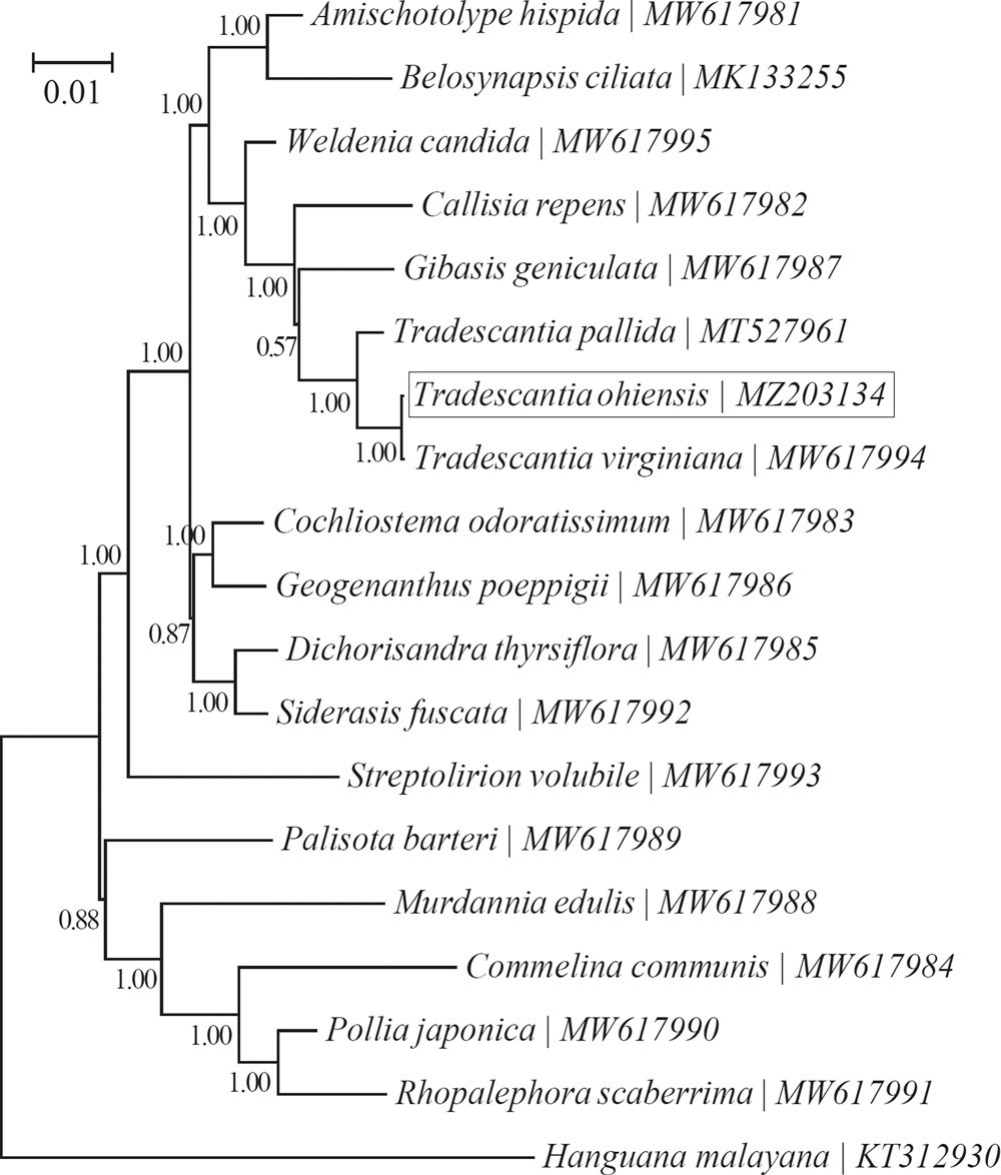
Phylogeny for Commelinaceae based on the Bayesian analysis of the concatenated coding sequences of chloroplast PCGs. The best-fit nucleotide substitution model is ‘GTR + G+I.’ The support values were placed next to the nodes. *Hanguana malayana* (Jack) Merr. (KT312930) was included as the outgroup taxon.

In this study, the complete cp genome was characterized for *T. ohiensis* with a discussion of its phylogenetic placement. The resultant cp genome sequence and associated high-throughput sequencing data would facilitate the development of molecular markers and contribute to the genetic assays of this exotic plant.

## Data Availability

The genome sequence data that support the findings of this study are openly available in GenBank of NCBI at [https://www.ncbi.nlm.nih.gov] under the accession number MZ203134. The associated **BioProject**, **SRA** and **Bio-Sample** numbers are PRJNA729969, SRR14540178 and SAMN19189328, respectively.
